# Similar Prevalence of *Plasmodium falciparum* and Non–*P. falciparum* Malaria Infections among Schoolchildren, Tanzania^1^

**DOI:** 10.3201/eid2906.221016

**Published:** 2023-06

**Authors:** Rachel Sendor, Cedar L. Mitchell, Frank Chacky, Ally Mohamed, Lwidiko E. Mhamilawa, Fabrizio Molteni, Ssanyu Nyinondi, Bilali Kabula, Humphrey Mkali, Erik J. Reaves, Naomi Serbantez, Chonge Kitojo, Twilumba Makene, Thwai Kyaw, Meredith Muller, Alexis Mwanza, Erin L. Eckert, Jonathan B. Parr, Jessica T. Lin, Jonathan J. Juliano, Billy Ngasala

**Affiliations:** University of North Carolina, Chapel Hill, North Carolina, USA (R. Sendor, C.L. Mitchell, T. Kyaw, M. Muller, A. Mwanza, J.B. Parr, J.T. Lin, J.J. Juliano);; National Malaria Control Programme, Dodoma, Tanzania (F. Chacky, A. Mohamed);; Muhimbili University of Health and Allied Sciences, Dar es Salaam, Tanzania (L.E. Mhamilawa, T. Makene, B. Ngasala);; Swiss Tropical and Public Health Institute, Basel, Switzerland (F. Molteni);; RTI International, Dar es Salaam (S. Nyinondi, B. Kabula, H. Mkali);; US Centers for Disease Control and Prevention, Dar es Salaam (E.J. Reaves);; US Agency for International Development, Dar es Salaam (N. Serbantez, C. Kitojo);; RTI International, Washington, DC, USA (E.L. Eckert);; Uppsala University, Uppsala, Sweden (B. Ngasala)

**Keywords:** malaria, Plasmodium falciparum, Plasmodium malariae, Plasmodium ovale, Plasmodium vivax, PCR, parasites, vector-borne infections, zoonoses, epidemiology, school survey, children, Tanzania

## Abstract

Achieving malaria elimination requires considering both *Plasmodium* *falciparum* and non–*P. falciparum* infections. We determined prevalence and geographic distribution of 4 *Plasmodium* spp. by performing PCR on dried blood spots collected within 8 regions of Tanzania during 2017. Among 3,456 schoolchildren, 22% had *P. falciparum,* 24% had *P. ovale* spp., 4% had *P. malariae*, and 0.3% had *P. vivax* infections*.* Most (91%) schoolchildren with *P. ovale* infections had low parasite densities; 64% of *P. ovale* infections were single-species infections, and 35% of those were detected in low malaria endemic regions. *P. malariae* infections were predominantly (73%) co-infections with *P. falciparum.* *P. vivax* was detected mostly in northern and eastern regions. Co-infections with >1 non–*P. falciparum* species occurred in 43% of *P. falciparum* infections. A high prevalence of *P. ovale* infections exists among schoolchildren in Tanzania, underscoring the need for detection and treatment strategies that target non–*P. falciparum* species.

Sub-Saharan Africa harbors 95% of the global malaria burden (*1*). National surveys conducted by ministries of health throughout Africa regularly assess *Plasmodium* *falciparum* prevalence (*2*); however, little is known about the prevalence and geographic distribution of non–*P. falciparum* (hereafter nonfalciparum) malaria species, such as *P*. *malariae*, *P*. *vivax*, and *P*. *ovale curtisi* or *P. ovale wallikeri* (hereafter *P*. *ovale*) (*3*–*8*). Although the clinical prevalence of nonfalciparum malaria in sub-Saharan Africa is dwarfed by *P. falciparum* (*9*), nonfalciparum species can still cause disease. *P*. *malariae* has been associated with increased risk for anemia (*10*) and other complications, such as chronic nephrotic syndrome (*11*,*12*). *P*. *vivax* can cause severe anemia, pregnancy-related complications, and death after recurrent infections, but infections in sub-Saharan Africa are infrequent (*13*–*15*). Clinical consequences of *P*. *ovale* infections have been mostly described in travelers and have been associated with severe infection in case reports (*16*).

Declining *P. falciparum* prevalence in East Africa might be associated with increasing nonfalciparum infections (*17*–*20*). However, comprehensive surveys of nonfalciparum malaria in sub-Saharan Africa have been infrequent because detection of those species remains challenging (*11*,*17*). Field diagnostic methods, such as microscopy and pan–*Plasmodium* spp. lactate dehydrogenase (LDH) or histidine-rich protein 2 (HRP2)–based rapid diagnostic tests (RDTs), lack sensitivity to detect nonfalciparum species (*11*,*17*). Nonfalciparum malaria parasite densities are often low, and most infected persons might not seek care. Mixed infections with *P. falciparum* can also complicate detection of nonfalciparum species (*3*,*17*). Molecular detection methods can sensitively detect nonfalciparum malaria species, but those methods remain largely confined to research use.

In Tanzania, the prevalence of malaria is high, accounting for 4.1% of global malaria deaths in 2020 (*1*). Although ≈93% of the population in mainland Tanzania is at risk for malaria, transmission throughout the country is highly heterogeneous (*21*). Transmission patterns are largely driven by geographic features of the country. Malaria transmission is low, unstable, and seasonal across the arid highlands and in urban centers; moderate and seasonally variable in southern, northern, and northwestern areas; and high and perennial along the coastal, lake, and southern lowland regions (*21*,*22*). Decades of concentrated malaria control interventions helped lower the national prevalence from 18% in 2008 to 7% in 2017 (*23*). Most reported malaria cases in Tanzania have been attributed to *P. falciparum* (*9*,*21*), but recent studies have also identified *P*. *malariae*, *P*. *vivax*, and *P*. *ovale* transmission (*4*,*18*,*24*,*25*). Given the widespread use of *P. falciparum*–specific HRP2-based RDTs for malaria diagnosis, the propensity for missed detection or misclassification of nonfalciparum species in Tanzania is high, and large-scale, geographically representative studies to assess spatial distributions of nonfalciparum malaria species are lacking. We used molecular methods to analyze blood samples collected during a national survey of schoolchildren in Tanzania and comprehensively characterize nonfalciparum malaria epidemiology. 

## Materials and Methods

### Study Design

The 2017 School Malaria Parasitological Survey (SMPS) was a cross-sectional study of children who were 5–16 years of age and enrolled in public primary schools in mainland Tanzania. Methods for site selection and survey design mirrored the 2015 SMPS and have been previously described (*22*). Study regions were selected through a multistage sampling scheme to maintain geographic representation and reflect the heterogeneity of malaria transmission across Tanzania (*22*,*26*). The number of schools randomly selected per region was proportional to each region’s respective population (*22*,*26*). Within each school, an average of 100 students were randomly selected for screening. After consent, each student was interviewed to obtain demographic and clinical characteristics, a malaria RDT was performed, and a dried blood spot (DBS) sample was collected (*22*,*26*). The survey largely coincided with each region’s rainy season. From among students who provided a DBS, we selected a stratified random subpopulation for nonfalciparum malaria testing. To maintain representativeness, we selected students in proportions that equaled regional proportions reflected within the broader survey population. 

Informed consent had been obtained from students and their legal guardians before survey data or blood sample collection, and ethical clearance was given by the Tanzania National Institute for Medical Research. Analysis of de-identified samples was approved by the Institutional Review Board of the University of North Carolina, Chapel Hill (approval no. 19-1495).

During the survey, malaria detection was conducted by using CareStart Malaria Pf/PAN (HRP2/pLDH) Ag Combo RDTs (AccessBio, https://www.accessbio.net) that were specific for *P. falciparum* HRP2 and pan-pLDH antigens. RDTs were considered positive if they were positive for either antigen. Schools and councils were grouped into epidemiologic malaria transmission risk strata on the basis of *P. falciparum* prevalences in children estimated from the 2014–15 Tanzania SMPS (*22*,*26*). *P. falciparum* prevalence was defined as very low if <5%, low if 5 to <10%, moderate if 10 to <50%, and high if >50% (*22*,*26*). DBS samples collected on Whatman filter paper (Cytiva, https://www.cytivalifesciences.com) were shipped to the University of North Carolina (Chapel Hill, NC, USA) for molecular testing.

### Molecular Detection

We extracted DNA from three 6-mm punches from each DBS sample by using a Chelex method (*27*) and performed real-time PCR targeting the 18S rRNA subunit of malaria as previously described (*28*) (Appendix Table 1). We performed PCR for each *Plasmodium* spp. independently with appropriate controls. We prepared positive controls for *P. falciparum* detection by using whole human blood and cultured *P. falciparum* strain 3D7 parasites (BEI Resources, https://www.beiresources.org) to create mock DBS samples and for nonfalciparum species detection by using plasmid DNA (BEI Resources). We serially diluted the control samples and extracted DNA as described. We estimated semiquantitative parasitemias for nonfalciparum species by assuming 6 18S rRNA gene copies/parasite (*28*) and multiplying by 4.0 to account for the 4-fold dilution of blood: ≈26 µL blood from 3 DBS punches (29) in 100 µL final volume of eluted DNA. We performed 40 PCR cycles for *P. malariae* and *P. falciparum* and 45 PCR cycles for *P. ovale* and *P. vivax* to enable detection of low-density infections (*28*). We previously validated this approach by using 390 negative controls comprising water (n = 22) and human DNA (n = 368) and >170 positive controls with decreasing nonfalciparum parasite densities; no false-positives were detected (*28*). We assessed PCR specificity by testing against 10 controls from each of the other *Plasmodium* spp.; no false positives were detected (Appendix Table 2). Our laboratory at the University of North Carolina participates in the World Health Organization malaria molecular quality assurance scheme, identifying and determining *Plasmodium* spp. in blinded samples every 6 months, and has consistently achieved high marks for assay performance across species. In this study, we did not detect false-positive amplification among 20 negative controls per each species-specific assay (Appendix Table 3). We performed further real-time PCR on a subset of *P. ovale*–positive samples to distinguish between *P. ovale wallikeri* and *P. ovale curtisi* (*30*,*31*). To evaluate potential bias from differences in PCR cycle numbers between species, we conducted a sensitivity analysis of randomly selected students (n = 750) stratified by malaria transmission risk. We performed semiquantitative real-time PCR of the 18S rRNA gene to 45 cycles to detect *P. falciparum* and *P. malariae* infections.

### Analysis

We calculated overall malaria species-specific prevalences and prevalence of single- and mixed-species infections. We did not adjust prevalences for sampling weight because nonfalciparum samples were selected randomly and in equal proportion to the broader survey sample.

We performed descriptive statistical analyses of student characteristics according to *Plasmodium* spp. We analyzed differences between *P. falciparum* and nonfalciparum single-species infections by using Pearson χ^2^ and Kruskal-Wallis rank-sum tests assuming nonnormality and applied Fisher exact test for small frequency counts. We performed similar analyses to compare malaria-positive and -negative students according to *Plasmodium* spp. Missing data were summarized, but we performed analyses on nonmissing data only. 

### Spatial Mapping

**Figure 6:**
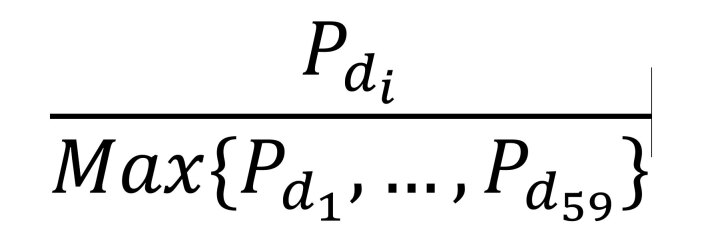
Equation.

We assessed regional variation in prevalence of each species through geospatial mapping by council and region. We aggregated numbers of infections and students by council and estimated and mapped council-level prevalences for each species. We calculated scaled prevalences by dividing the proportion of each council’s prevalence by the highest council prevalence for each *Plasmodium* species, as follows (Figure 6):

where *P* is the prevalence for a given council, *d_i_*. We calculated and mapped differences between scaled nonfalciparum and scaled *P. falciparum* prevalences for each council. This method compared prevalence estimates between each nonfalciparum species and *P. falciparum*, while accounting for differences in the absolute burden of each species.

We performed analyses by using R version 4.0.2 (The R Project for Statistical Computing, https://www.r-project.org) and used the eulerr (https://cran-r-project.org/package=eulerr) and sf version 0.9–7 (*32*) packages for prevalence visualization and mapping. We sourced shapefiles from the Global Administrative Areas database (https://gadm.org) and collected elevation measurements from the US National Aeronautics and Space Administration, Shuttle Radar Topography Mission (https://www.nasa.gov). 

## Results

### Study Population

We selected a total of 3,456 students from 180 schools across 8 geographic regions for nonfalciparum malaria testing from among 17,131 students in the SMPS who had available DBS samples. We did not detect differences in student characteristics between those in the nonfalciparum malaria and SMPS DBS populations (Appendix Table 4). Median (interquartile range [IQR]) student age in the nonfalciparum study population was 11 (9–13) years; distribution of male (51%) and female (49%) students was similar. Malaria dual-antigen RDTs were positive in 20% of students. Most students attended schools in regions classified as high (51%) or moderate (13%) malaria transmission risk (Table 1).

**Table 1:** Characteristics of students infected by different *Plasmodium* spp. in study of similar prevalence of *Plasmodium falciparum* and non–*P. falciparum* malaria infections among schoolchildren, Tanzania*

Characteristics	Single species infections							*P*. *falciparum* co-infections				Other§	Total
	Pf	Po	p value†	Pm	p value‡	Pv		Po+Pf	Pm+Pf	Po+Pm+Pf	Pv+Pf		
No. students	429 (12.4)	519 (15.0)		24 (0.7)				224 (6.5)	44 (1.3)	55 (1,6)	2 (0.1)	NA	3,456
Median age, y (IQR)	12 (10–13)	11 (9–12)	<0.001	11 (10–13)	0.821	11 (10–11)		12 (9–14)	11 (9–12)	12 (10–13)	12 (12–13)	12 (10–12)	11 (9, 13)
Sex													
M	236 (55.0)	240 (46.2)	0.009	15 (62.5)	0.612	3 (75.0)		120 (53.6)	27 (61.4)	34 (61.8)	2 (100.0)	9 (52.9)	1,761 (51.0)
F	193 (45.0)	279 (53.8)		9 (37.5)		1 (25.0)		104 (46.4)	17 (38.6)	21 (38.2)	0 (0.0)	8 (47.1)	1,695 (49.0)
Fever¶	40 (10.8)	8 (2.1)	<0.001	3 (15.8)	0.455	0 (0.0)		12 (6.1)	6 (16.7)	2 (4.7)	0 (0.0)	1 (6.2)	99 (3.5)
Missing data	60	134		5		1		28	8	12	0	1	618
Malaria RDT+	295 (68.8)	40 (7.7)	<0.001	8 (33.3)	0.001	1 (25.0)		170 (76.9)	36 (83.7)	45 (81.8)	2 (100.0)	9 (52.9)	686 (19.9)
Missing tests	0	0		0		0		3	1	0	0	0	15
Epidemiologic risk strata#													
High	367 (85.5)	293 (56.5)	<0.001	19 (79.2)	0.484	3 (75.0)		204 (91.1)	41 (93.2)	53 (96.4)	2 (100.0)	16 (94.1)	1,768 (51.2)
Moderate	49 (11.4)	45 (8.7)		5 (20.8)		1 (25.0)		16 (7.1)	3 (6.8)	1 (1.8)	0	1 (5.9)	448 (13.0)
Low	9 (2.1)	155 (29.9)		0		0		4 (1.8)	0	1 (1.8)	0	0	602 (17.4)
Very low	4 (0.9)	26 (5.0)		0		0		0	0	0 (0.0)	0	0	638 (18.5)

*Values are no. (%) students unless otherwise indicated. Percentages were calculated according to the species-specific totals for nonmissing data. Species were determined by using real-time PCR. Continuous variables were compared by using Kruskal-Wallis test; categorical variables were compared by using χ^2^ test; and Fisher exact test was applied when cell counts were <5 cells/μL. IQR, interquartile range; Pf, *Plasmodium* *falciparum*; Pm, *P*. *malariae*; Po, *P*. *ovale* spp.; Pv, *P*. *vivax*; RDT, rapid diagnostic test; +, positive. †p value for Pf versus Po. ‡p value for Pf versus Pm. §Other mixed infections included Po + Pm (n = 12), Po + Pv (n = 3), Pm + Pv (n = 1), and Po + Pv + Pf (n = 1). Cases of Po + Pm + Pv, Pm + Pv + Pf, or Po + Pm + Pv + Pf co-infections were not detected. ¶Fever was defined as temperature >38°C at time of survey. Body temperature was missing for 618 (17.9%) students. #Epidemiologic risk strata are defined according to *P*. *falciparum* prevalences in children from the 2014–15 School Malaria Parasitological Survey, Tanzania: very low if prevalence <5%, low if 5% to <10%, moderate if 10% to <50%, and high if >50%.

### Species Prevalence Determined by PCR

**Figure 1:**
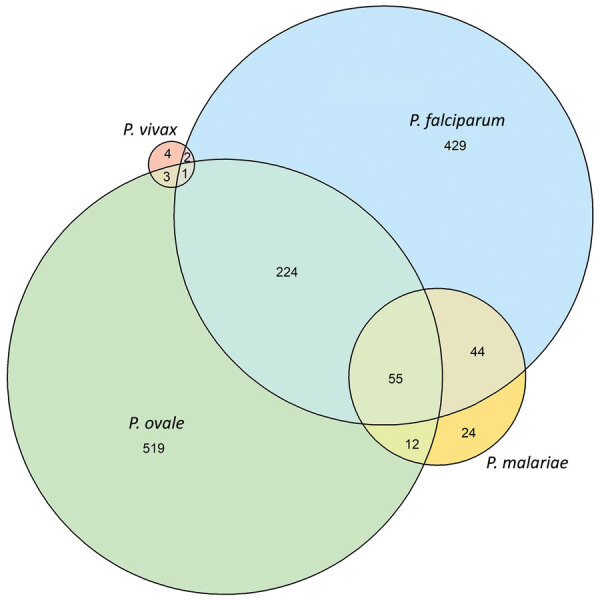
Distribution of *Plasmodium* spp. infections among schoolchildren, Tanzania. Prevalence estimates according to species: *P. falciparum*, 21.8% (95% CI 20.5%–23.3%, n = 755); *P. ovale*, 23.6% (95% CI 22.2%–25.0%, n = 814); *P. malariae*, 3.9% (95% CI 3.3%–4.6%, n = 136); *P. vivax*: 0.3% (95% CI 0.2%–0.6%, n = 11). *P. vivax + P. malariae* co-infection (n = 1) is not shown.

We identified *P. falciparum* infections in 22% (95% CI 21%–23%, n = 755), *P. ovale* in 24% (95% CI 22%–25%, n = 814), *P. malariae* in 4% (95% CI 3%–5%, n = 136), and *P. vivax* in 0.3% (95% CI 0.2%–0.6%, n = 11) of students, including single- and mixed-species infections (Appendix Table 5). Most (64%, n = 519) *P. ovale* infections were single-species infections; 28% (n = 224) were co-infections with *P. falciparum* only (Figure 1). Conversely, most (40%, n = 55) *P. malariae* infections were co-infections with both *P. ovale* and *P. falciparum*; 32% (n = 44) were co-infections with *P. falciparum* only. We determined 36% (n = 4) of *P. vivax* infections were single-species infections, and 43% (n = 326) of *P. falciparum* infections were co-infections with >1 nonfalciparum malaria species.

We conducted a sensitivity analysis, detecting *P. falciparum* and *P. malariae* by using PCR cycle thresholds of <45 to evaluate different PCR cycles between assays. We observed 25% (95% CI 21%–29%) *P. falciparum* and 3% (95% CI 2%–5%) *P. malariae* prevalences, weighted according to student distribution within the total nonfalciparum population by transmission risk strata (Appendix Table 6). Within that subset, 2.5% (n = 4) of *P. falciparum* and 10% (n = 2) of *P. malariae* infections were detected at cycle thresholds of 40–45. Thus, >97% of *P. falciparum* and 90% of *P. malariae* infections were detectable by the primary 40-cycle assay in our study.

We evaluated differences in student characteristics according to *Plasmodium* spp. infection (Table 1; Appendix Table 7). We detected *P. ovale* single-species infections more frequently than *P. falciparum* infections in slightly younger (median 11 vs. 12 years of age; p<0.001) and female (54% vs. 45%; p = 0.009) students. Comparing RDT sensitivity to PCR, we observed 8% (n = 40) of students with *P. ovale* single-species infections were RDT-positive for any band, whereas 33% (n = 8) of those with *P. malariae* and 69% (n = 295) with *P. falciparum* single-species infections were RDT-positive. Co-infections with *P. falciparum* and nonfalciparum were RDT-positive in 78% (n = 253/325) of cases detected by PCR. Although only 3% (n = 13) of *P. falciparum* single-species infections and no *P. malariae* or *P. vivax* single-species infections were detected in low transmission risk strata, 35% (n = 181) of *P. ovale* single-species infections occurred in regions classified as low or very low malaria transmission risk. High epidemiologic risk strata harbored most single-species infections across all 4 *Plasmodium* spp. and also mixed infections with *P. falciparum*.

### Parasite Density

**Figure 2:**
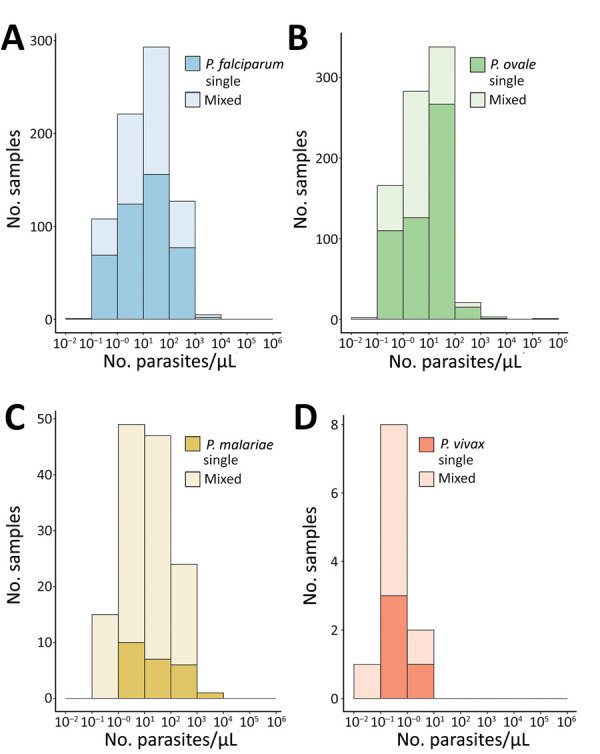
Estimated parasite density distributions according to malaria species in study of similar prevalence of *Plasmodium falciparum* and non–*P. falciparum* malaria infections among schoolchildren, Tanzania. We estimated *Plasmodium* spp. parasite densities for single infections and co-infections (mixed) by using semiquantitative PCR and species-specific primers (Appendix Table 1). Mixed infections included *P. falciparum* and nonfalciparum co-infections. Number of samples varied by species. *P. ovale* and *P. vivax* parasite densities were detected by using 45 PCR cycles; other species were determined by using 40 PCR cycles. A) *P. falciparum*: median (IQR) density was 11.4 (2.5–54.7) parasites/µL for single-species infections (n = 429) and 16.5 (3.5–56.9) parasites/µL for mixed-species infections (n = 326) (p = 0.117). B) *P. ovale*: median (IQR) density was 13.5 (1.3–30.1) parasites/µL for single-species infections (n = 519) and 3.1 (1.2–11.4) parasites/µL for mixed-species infections (n = 295) (p<0.001). C) *P. malariae*: median (IQR) density was 16.1 (3.8–164.0) parasites/µL for single-species infections (n = 24) and 11.2 (2.6–53.9) parasites/µL for mixed-species infections ([n = 112) (p = 0.169). D) *P. vivax*: median (IQR) density was 0.4 (0.2–0.9) parasites/µL for single-species infections (n = 4) and 0.7 (0.5–0.8) parasites/µL for mixed-species infections (n = 7) (p = 0.571). IQR, interquartile range.

Malaria parasitemia estimated by semiquantitative PCR was low across nonfalciparum species (Figure 2). Median (IQR; min–max) *P. ovale* density was 7.2 (1.3–25.0; 0.1–168,596) parasites/µL, comparable to *P. malariae* density at 11.7 (2.7–54.9; 0.3–1,214) parasites/µL. *P. vivax* density was ≈0.6 (0.3–0.8; 0.1–8.1) parasites/µL. Although 18% (n = 25) of *P. malariae* infections had a parasite density >100 parasites/µL, we rarely observed that level for *P. ovale* (3%, n = 25) and never for *P. vivax*. *P. falciparum* density was also low at 13.1 (2.6–55.9; 0.1–8,248) parasites/µL; however, 17% (n = 132) of *P. falciparum* cases had a parasite density >100 parasites/µL, and 3% (n = 24) had >500 parasites/µL. Median (IQR) density among *P. ovale* mixed infections was 3.1 (1.2–11.4) parasites/µL and 13.5 (1.3–30.1) parasites/µL for *P. ovale* single-species infections (p<0.001), whereas densities were similar between single- and mixed-species infections among the other malaria species (Figure 2).

### *P. ovale* Species Determination

Among 814 samples positive for *P. ovale*, 60 (7%) samples with the highest parasitemia were selected for PCR to distinguish between *P. ovale wallikeri* and *P. ovale curtisi*. Species determination by PCR was successful in 35% (n = 21) of samples; *P. ovale curtisi* was detected in 17 samples and *P. ovale wallikeri* in 9 samples. We identified *P. ovale curtisi* and *P. ovale wallikeri* co-infections in 5 students. We did not perform further characterization because of limited sample sizes.

### Geographic Distribution

**Figure 3:**
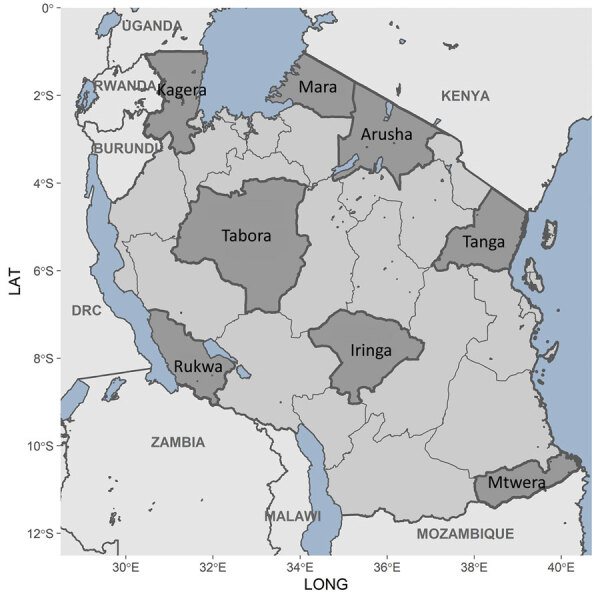
Locations of 8 survey regions within mainland Tanzania (dark gray shading) in study of similar prevalence of *Plasmodium falciparum* and non–*P. falciparum* malaria infections among schoolchildren, Tanzania.

**Figure 4:**
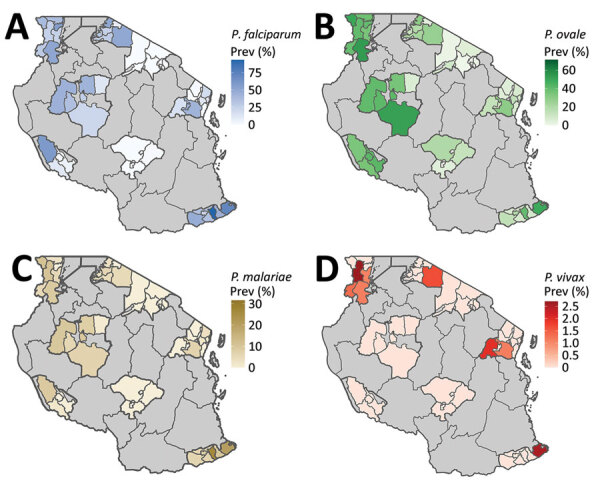
Spatial distribution of regional and school council–level malaria prevalence by species in study of similar prevalence of *Plasmodium falciparum* and non–*P. falciparum* malaria infections among schoolchildren, Tanzania. A) *P. falciparum*; B) *P. ovale*; C) *P. malariae*; D) *P. vivax*.

We detected *P. ovale* across all 8 regions sampled in Tanzania, indicating widespread distribution (Table 2; Figures 3, 4). *P. ovale* prevalence was highest within the northern Kagera (34%, n = 273) and central Tabora (17%, n = 139) regions. We detected *P. ovale curtisi* infections in 6 of 8 regions (all but Arusha and Rukwa) and *P. ovale wallikeri* in 5 of 8 (Kagera, Mara, Tabora, Tanga, and Iringa) regions. We observed high prevalence of *P. malariae* in Kagera (29%, n = 39) and in southernmost Mtwara (28%, n = 38), and *P. vivax* was predominantly distributed along the northwestern borders of Tanzania in Kagera (55%, n = 6); select, isolated cases of *P. vivax* were also detected in southern and eastern regions. Arusha and Iringa did not have any cases of *P. malariae* or *P. vivax* infections and had the lowest frequencies of *P. ovale* (3%, n = 23, in Arusha; 4%, n = 30, in Iringa) and *P. falciparum* (0.4%, n = 3, in Arusha; 0.1%, n = 1, in Iringa) infections.

**Table 2:** Number of students infected with *Plasmodium* spp. and school characteristics in study of similar prevalence of *Plasmodium falciparum* and non–*P. falciparum* malaria infections among schoolchildren, Tanzania*

School characteristics‡	*Plasmodium* spp. infections†				Total, n = 3,456
	Pf, n = 755	Po, n = 814	Pm, n = 136	Pv, n = 11	
Elevation, m					
Median (IQR)	1,182 (506–1,370)	1,225 (1,124–1,427)	1,167 (320–1,370)	1,333 (1,100–1,398)	1,230 (1,058–1,467)
Minimum–maximum	54–1,901	47–2,167	54–1,677	184–1,467	34–2,167
<1,500	707 (26.5)	693 (26.0)	129 (4.8)	11 (0.4)	2,667 (100)
>1,500	48 (6.1)	121 (15.3)	7 (0.9)	0	789 (100)
Region‡					
Arusha	3 (0.5)	23 (4.2)	0	0	552 (100)
Iringa	1 (0.3)	30 (9.4)	0	0	320 (100)
Kagera	196 (31.7)	273 (44.1)	39 (6.3)	6 (1.0)	619 (100)
Mara	157 (34.7)	102 (22.6)	19 (4.2)	2 (0.4)	452 (100)
Mtwara	146 (47.6)	62 (20.2)	38 (12.4)	1 (0.3)	307 (100)
Rukwa	49 (16.3)	118 (39.2)	6 (2.0)	0	301 (100)
Tabora	122 (29.5)	139 (33.7)	25 (6.1)	0	413 (100)
Tanga	81 (16.5)	67 (13.6)	9 (1.8)	2 (0.4)	492 (100)

*Values are no. (%) students unless otherwise indicated. IQR, interquartile range; Pf, *Plasmodium* *falciparum;* Pm, *P*. *malariae*; Po, *P*. *ovale* spp.; Pv, *P*. *vivax.*†Totals and percentages in each species-specific column represent student-level prevalences and include single- and mixed-species infections. ‡Listed regions correspond to mapped regions in Figure 3.

We detected malaria infections in students who were predominantly located at elevations <1,500 m, including 85% (n = 693) infected by *P. ovale*, 94% (n = 707) by *P. falciparum,* 95% (n = 129) by *P. malariae*, and 100% (n = 11) by *P. vivax* (Table 2). Most (77%, n = 2,667) students enrolled in our study were from schools located at elevations <1,500 m. Among students located at elevations >1,500, *P. ovale* infections were detected most frequently in 15% (n = 121) of students compared with 6% (n = 48) infected with *P. falciparum*, 1% (n = 7) infected with *P. malariae*, and 0% infected with *P. vivax*.

**Figure 5:**
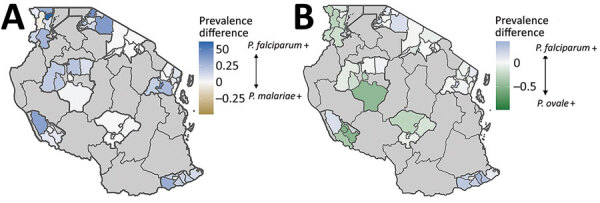
Differential scaled prevalences between *Plasmodium malariae* or *P. ovale* and *P. falciparum* at the school council level in study of similar prevalence of *Plasmodium falciparum* and non–*P. falciparum* malaria infections among schoolchildren, Tanzania. A) Blue shading indicates councils where *P. falciparum* scaled prevalence is greater (indicated by + in key) than *P. malariae* scaled prevalence; gold indicates regions where *P. malariae* scaled prevalence is greater. B) Light blue shading indicates councils where *P. falciparum* scaled prevalence is greater than *P. ovale* spp. scaled prevalence; green indicates regions where *P. ovale* scaled prevalence is greater. Comparison of scaled prevalences for *P. falciparum* and *P. vivax* is not depicted because the low number of *P. vivax* infections biased the scaled measurement.

We compared scaled prevalence estimates for nonfalciparum species with *P. falciparum* and identified areas where prevalences were higher than expected for *P. ovale* and *P. malariae* on the basis of *P. falciparum* frequency (Figure 5); *P. vivax* infections were too infrequent for comparison. In the southern and southwestern highlands and northwestern lake regions (Iringa, Rukwa, Tabora, and Kagera), scaled *P. ovale* prevalences were higher than *P. falciparum* prevalences. Scaled prevalence of *P. malariae* was notably higher than that of *P. falciparum* in the Karagwe council in Kagera and Mtwara municipal council in Mtwara. In most other areas, scaled prevalence of *P. malariae* was similar to or lower than *P. falciparum* prevalence.

## Discussion

Our study describes a large nationally representative molecular survey of nonfalciparum malaria epidemiology across Tanzania. We used real-time PCR to estimate nonfalciparum infection prevalences in school-aged children in 8 regions of the country selected to maintain geographic diversity and malaria transmission risk heterogeneity. One quarter (24%) of schoolchildren harbored *P. ovale* parasites, comparable to the 22% *P. falciparum* prevalence in the population, and 64% of *P. ovale* infections were single-species infections. *P. malariae* was observed in 4% of students, of which most were co-infected with other malaria species. *P. vivax* infections were rare (0.3% prevalence).

High *P. ovale* prevalence could be attributed to several factors. First, we increased the number of PCR cycles for *P*. *ovale* detection to 45 to enable detection of low-density infections, which comprised 91% of all *P. ovale* infections identified (Appendix Table 8). This approach has precedence (*17*,*25*,*31*), as low-density parasitemia is characteristic of *P. ovale* infections, making detection challenging. Using 40-cycle PCR for *P. ovale* yielded a 0.8% prevalence estimate in our previous work in the Democratic Republic of the Congo (*5*). The prevalence of *P. ovale* infections positive at <40 cycles in this study was 9% (n = 75), confirming most infections occurred at very low parasite densities. Second, many large-scale molecular surveys of nonfalciparum malaria have focused on adults or all-age cohorts, whereas school-aged children are increasingly recognized as the main contributors to asymptomatic and infectious malaria reservoirs (*33*–*35*). Finally, the high prevalence of *P. ovale* in our study might reflect increasing *P. ovale* transmission despite malaria control efforts targeting *P. falciparum*. Increasing or persistent transmission of *P. ovale* and *P. malariae* amid a *P. falciparum* decline has been observed in molecular surveys from Tanzania and nearby Kenya and Uganda, including in symptomatic cases (*17*,*18*,*24*,*36*). The causes of increased transmission are unclear but might include hypnozoite-induced relapses of *P. ovale* infections not treated by artemisinin-based combination therapies, insect day-biting, or outdoor vectors that evade bed nets.

In contrast to findings from other studies (*11*,*36*–*39*), we found that *P. ovale* infections occurred more commonly as single-species infections than did other nonfalciparum species infections, although increased sensitivity of *P. ovale*–specific PCR might partially explain those observations. *P. ovale* single-species infections were rarely detected by RDTs, rendering them more difficult to detect and treat. In addition, *P. ovale* single-species infections were largely the only infections identified within regions categorized as low risk for malaria transmission, suggesting an unexpected transmission risk in areas where prevention measures might be less common and *P. falciparum* risk is not a particular concern. Our scaled differential prevalence map similarly highlighted several councils where *P. ovale* and *P. malariae* prevalences were proportionally higher than expected on the basis of *P. falciparum* frequency. Taken together, those characteristics indicate a hidden burden of *P. ovale* infections in Tanzania.

Detection of *P. vivax* in this study is notable given the infection control challenges posed by this species. Infections were predominately detected in the northwest/Lake regions of Tanzania and in the east, where several other studies have also observed low *P. vivax* prevalences (*4*,*24*,*40*). *P. malariae* prevalence of 4% aligns with recent research in the region that also identified low infection prevalences (2.5% in Malawi, 4.1% in Democratic Republic of the Congo, and 3.3% symptomatic and 5.3% asymptomatic cases in western Kenya) (*12*,*28*,*39*). Estimated parasite densities were low across nonfalciparum species, as expected. *P. falciparum* parasite densities were also relatively low (median 13.1 parasites/µL), likely because of the predominantly asymptomatic population. In addition, mapping confirmed low or nonexistent prevalence of nonfalciparum malaria within the northern highlands of Arusha and southern highlands and midlands of Iringa.

The first limitation of our study is that using different PCR cycling times for different species introduces ascertainment bias. Because *P. malariae* and *P. falciparum* assays were run at 40 rather than 45 cycles, their relative prevalences compared with prevalence for *P. ovale* might be underestimated. However, we performed a sensitivity analysis to quantify this bias, which indicated that only an additional 2.5% of *P. falciparum* and 10% of *P. malariae* infections would be detected by using 45 cycles, suggesting minimal underestimation of reported *P. falciparum* and *P. malariae* prevalences and no meaningful effect on overall conclusions. Weighting sensitivity analysis results to the total study population yielded a *P. falciparum* prevalence of 25% if 45 cycles were used compared with the observed prevalence of 22%. Despite this result, prevalences could still be underestimated given lower probabilities of detecting very low density infections because of PCR limits of detection in concert with small volumes of template DNA used in the assays (2 µL for *P. malariae, P. ovale*, and *P. falciparum*; 5 µL for *P. vivax*). Second, our study did not sample all geographic regions in Tanzania, and findings cannot be extrapolated to other age groups with differing malaria risk profiles. School-based sampling likely underestimated prevalence of symptomatic or severe malaria infection in school-aged children because children might have been absent because of illness. Finally, the cross-sectional survey design revealed little about clinical implications of prevalent nonfalciparum infections, especially given substantial nonrandom missingness in fever data, or the extent to which infections represented chronic infection carriage versus transient parasitemia.

In conclusion, the overall high prevalence and broad geographic distribution of *P. ovale* and, to a lesser extent, *P*. *malariae* and the more focal distribution of *P. vivax* in this study underscore an urgent need to elucidate clinical prevalence and transmission patterns of those species to inform malaria control programs in Tanzania. Current treatment protocols in Tanzania do not regularly address hypnozoite liver-stage *P. ovale* infection, and relapses are expected after blood-stage clearance by artemisinin-based combination therapy (*41*). Accumulating evidence exists for increases in previously unappreciated nonfalciparum malaria infections in sub-Saharan Africa (*38*). Molecular detection methods, such as PCR, and new treatment strategies will be required for continued progress toward malaria control and elimination.

Appendix Additional information for similar prevalence of Plasmodium falciparum and non–P. falciparum malaria infections among schoolchildren, Tanzania.
