# Supplementary material for: Similar Prevalence of Plasmodium falciparum and Non–P. falciparum Malaria Infections among Schoolchildren, Tanzania
Source: Emerg Infect Dis. 2023 Jun;29(6):1143–53. doi: 10.3201/eid2906.221016 (PMC10202886; doi:10.3201/eid2906.221016)
Supplement: Appendix — Additional information for similar prevalence of Plasmodium falciparum and non–P. falciparum malaria infections among schoolchildren, Tanzania. [file 22-1016-Techapp-s1.pdf]

Article DOI: https://doi.org/10.3201/eid2906.221016

EID cannot ensure accessibility for supplementary materials supplied by authors. Readers who have difficulty accessing supplementary content should contact the authors for assistance.

Similar Prevalence of *Plasmodium falciparum* and Non–*P. falciparum* Malaria Infections among Schoolchildren, Tanzania

Appendix

Appendix Table 1. PCR primers and probes used to detect *Plasmodium* spp.

Plasmodium sp.	Oligonucleotides	nmol/L*	Sequence (5′–3′)
P. malariae	Forward primer	300	AGTTAAGGGAGTGAAGACGATCAGA
	Reverse primer	300	CAACCCAAAGACTTTGATTTCTCATAA
	Probe	200	FAM-ATGAGTGTTTCTTTTAGATAGC-MGBNFQ
P. ovale spp.	Forward primer	400	CCRACTAGGTTTTGGATGAAAVRTTTTT
	Reverse primer	400	AACCCAAAGACTTTGATTTCTCATAA
	Probe	200	VIC-CRAAAGGAATTYTCTTATT-MGBNFQ
P. vivax	Forward primer	400	ACGCTTCTAGCTTAATCCACATAACT
	Reverse primer	400	ATTTACTCAAAGTAACAAGGACTTCCAAGC
	Probe	200	FAM-TTCGTATCG/ZEN/ACTTTGTGCGCATTTTGC-3IABkFQ
P. falciparum	Forward primer	300	ATTGCTTTTGAGAGGTTTTGTTACTTT
	Reverse primer	300	GCTGTAGTATTCAAACACAATGAACTCAA
	Probe	200	FAM-CATAACAGACGGGTAGTCAT
P. ovale curtisi	Forward primer	300	TTTTGAAGAATACATTAGGATACAATTAATG
	Reverse primer	300	CATCGTTCCTCTAAGAAGCTTTACAAT
	Probe	200	HEX-CCTTTTCCC/ZEN/TATTCTACTTAATTCGCAATTCATG
P. ovale wallikeri	Forward primer	300	TTTTGAAGAATATATTAG-GATACATTATAG
	Reverse primer	300	CATCGTTCCTCTAAGAAGCTTTACAAT
	Probe	200	FAM-CCTTTTCCCTTTTCTACTTAATTCGCTATTCATG-TAMRA

*Final concentrations of primers and probes used to detect *Plasmodium* spp. by PCR.

Appendix Table 2. PCR specificity testing for nonfalciparum malaria species

	PCR results			
Plasmodium spp. tested	No. controls	No. positive	No. negative	% Specificity
P. ovale spp. tested against				
P. malariae	10	0	10	100
P. vivax	10	0	10	100
P. falciparum	10	0	10	100
P. malariae tested against				
P. ovale spp.	10	0	10	100
P. vivax	10	0	10	100
P. falciparum	10	0	10	100
P. vivax tested against				
P. ovale spp.	10	0	10	100
P. malariae	10	0	10	100
P. falciparum	10	0	10	100

Appendix Table 3. *Plasmodium* spp.–specific PCR control assays*

Plasmodium spp.	Parasites/µL	No. controls	No. positive†	% Positive	Mean Ct	SD‡
P. malariae						
Standard 1	10,000	20	20	100	23.7	0.64
Standard 2	1,000	20	20	100	27.4	0.69
Standard 3	100	20	20	100	31.1	0.96
Standard 4	10	20	20	100	34.5	1.44
Standard 5	1	20	6	30	37.4	1.47
Negative control	0	20	0	0	NA	NA
P. ovale spp.						
Standard 1	10,000	19	19	100	27.7	2.93
Standard 2	1,000	20	19	95	31.0	2.79
Standard 3	100	19	19	100	34.3	2.39
Standard 4	10	20	14	*70*	37.0	1.64
Standard 5	1	20	10	50	40.7	1.31
Negative control	0	20	0	0	NA	NA
P. vivax						
Standard 1	10,000	20	20	100	25.7	0.54
Standard 2	1,000	20	20	100	28.5	0.86
Standard 3	100	20	18	90	32.5	0.86
Standard 4	10	20	20	100	36.1	0.59
Standard 5	1	20	15	75	39.0	1.07
Negative control	0	20	0	0	NA	NA
P. falciparum						
Standard 1	10,000	20	20	100	20.4	0.46
Standard 2	1,000	20	20	100	24.4	0.29
Standard 3	100	20	20	100	27.7	0.35
Standard 4	10	20	19	95	31.7	0.26
Standard 5	1	20	10	50	35.5	0.84
Negative control	0	20	0	0	NA	NA

*Negative controls were water. Controls were run in duplicate in each PCR plate. Standards were plated manually after plating clinical samples by using an automated PCR robot. Ct, cycle threshold; NA, not applicable.
†PCR assays in which negative controls amplified incorrectly (e.g., because of contamination) were rerun until controls remained negative. Results from original assays were then discarded as invalid and the rerun assay results were used in final analyses.
‡Precision of *P. ovale* spp. standard Ct values were validated by using 320 additional *P. ovale* standards with varied concentrations; an automated PCR robot was used to plate standards because manual plating might have increased Ct variability across plates.

Appendix Table 4. Comparison of characteristics between students in the full SMPS cohort and students in the subcohort for nonfalciparum malaria analysis*

Student characteristics	Full SMPS cohort	Subcohort	p value†
Total no. students	17,131	3,456	NA
Median age (IQR), y	11 (9–13)	11 (9–13)	0.884
Male students	8,457 (49)	1761 (51)	0.093
Malaria rapid diagnostic tests	3,328 (20)	686 (20)	0.589
Median school elevation (IQR), m	1,230 (1,058–1,458)	1,230 (1,058–1,467)	0.925
Epidemiologic risk			0.966
High	8,806 (51)	1,768 (51)	NA
Moderate	2,180 (13)	448 (13)	NA
Low	2,952 (17)	602 (17)	NA
Very low	3,193 (19)	638 (18)	NA
Region			1.0
Arusha	2,757 (16)	552 (16)	NA
Iringa	1,583 (9)	320 (9)	NA
Kagera	3,078 (18)	619 (18)	NA
Mara	2,228 (13)	452 (13)	NA
Mtwara	1,511 (9)	307 (9)	NA
Rukwa	1,506 (9)	301 (9)	NA
Tabora	2,045 (12)	413 (12)	NA
Tanga	2,423 (14)	492 (14)	NA

*Values are no.(%) students unless otherwise indicated. IQR, interquartile range; NA, not applicable; SMPS, School Malaria Parasitological Survey from 2017.
†Continuous variables were compared by using a Kruskal-Wallis test. Categorical variables were compared by using χ^2 test.

Appendix Table 5. Prevalences and 95% CIs for *Plasmodium* spp.–specific malaria infections*

Plasmodium spp.	Point prevalence† No.	%	95% CI
Total student population	3,456	100.00	NA
Total infections‡			
Po	814	23.6	22.2–25.0
Pm	136	3.9	3.3–4.6
Pv	11	0.3	0.2–0.6
Pf	755	21.8	20.5–23.3
Single-species infections			
Po	519	15.0	13.9–16.3
Pm	24	0.7	0.5–1.0
Pv	4	0.1	0.1–0.3
Pf	429	12.4	11.4–13.6
Mixed species infections§			
P. falciparum co-infection			
Po + Pf	224	6.5	5.7–7.4
Pm + Pf	44	1.3	1.0–1.7
Pv + Pf	2	0.1	0.02–0.2
Po + Pm + Pf	55	1.6	1.2–2.1
Po + Pv + Pf	1	0.03	0.01–0.2
Pm + Pv + Pf	0	0	0
Po + Pm + Pv + Pf	0	0	0
Nonfalciparum species only			
Po + Pm	12	0.3	0.2–0.6
Po + Pv	3	0.1	0.03–0.3
Pm + Pv	1	0.03	0.01–0.2
Po + Pm + Pv	0	0	0

*NA, not applicable; Pf, *Plasmodium falciparum*; Po, P. *ovale* spp.; Pm, P. *malariae*; Pv, P. *vivax*.
†Percents are calculated on the basis of the total study population.
‡Total infections include both single- and mixed-species infections.
§Categories are mutually exclusive.

Appendix Table 6. Sensitivity analysis for *Plasmodium falciparum* and *P. malariae* prevalence in students estimated by using different real-time PCR cycle thresholds*

	Epidemiologic risk strata				Crude prevalence		Weighted prevalence†	
Plasmodium spp.	Low, n = 250	Moderate, n = 250	High, n = 250	Total, n = 750	%	95% CI	%	95% CI
P. falciparum								
PCR positive	5 (2.0)	47 (18.8)	105 (42.0)	157 (20.9)	20.9	17.8–24.5	24.6	20.6–29.2
Ct <40	5 (2.0)	45 (18.0)	103 (41.2)	153 (20.4)	NA	NA	NA	NA
Ct 40 to <45	0 (0.0)	2 (0.8)	2 (0.8)	4 (0.5)	NA	NA	NA	NA
PCR Negative	245 (98.0)	203 (81.2)	145 (58.0)	593 (79.1)	NA	NA	NA	NA
P. malariae								
PCR positive	2 (0.8)	6 (2.4)	12 (4.8)	20 (2.7)	2.7%	1.6–4.1	3.1	1.8–4.9
Ct <40	1 (0.4)	6 (2.4)	11 (4.4)	18 (2.4)	NA	NA	NA	NA
Ct 40 to <45	1 (0.4)	0 (0.0)	1 (0.4)	2 (0.3)	NA	NA	NA	NA
PCR Negative	248 (99.2)	244 (97.6)	238 (95.2)	730 (97.3)	NA	NA	NA	NA

*Values are no. (%) students unless otherwise noted. Real-time PCR targeted the 18S rRNA gene. Sensitivity analysis population was a stratified random sample of the study population; sampling was stratified by epidemiologic malaria risk to maintain malaria transmission heterogeneity within the sample. Low epidemiologic risk classification includes very low and low risk strata. Ct, cycle threshold; NA, not applicable.
†Prevalence estimates were weighted according to student distribution within the study population and epidemiologic malaria risk strata to account for oversampling of the moderate risk strata.

Appendix Table 7. Characteristics of students with or without nonfalciparum malaria infections identified by PCR*

	P. ovale spp.			*P. malariae*			*P. vivax*		
	PCR+	PCR–		PCR+	PCR–		PCR+	PCR–	
Characteristics	Any Infection	No infection	p value	Any Infection	No infection	p value	Any Infection	No infection	p value
No. students	814	2,138	NA	136	2,138	NA	11	2,138	NA
Median age (IQR), y	11 (9–13)	11 (9–13)	0.128	12 (9–13)	11 (9–13)	0.038	11 (9.5–12)	11 (9–13)	0.95
Male students	402 (49.4)	1,075 (50.3)	0.694	83 (61.0)	1,075 (50.3)	0.019	8 (72.7)	1,075 (50.3)	0.226
Fever†	23 (3.6)	27 (1.5)	0.003	12 (10.9)	27 (1.5)	<0.001	0 (0.0)	27 (1.5)	1
Epidemiologic risk strata‡			<0.001			<0.001			0.001
High	565 (69.4)	770 (36.0)	NA	125 (91.9)	770 (36.0)	NA	10 (90.9)	770 (36.0)	NA
Moderate	63 (7.7)	327 (15.3)	NA	10 (7.4)	327 (15.3)	NA	1 (9.1)	327 (15.3)	NA
Low	160 (19.7)	433 (20.3)	NA	1 (0.7)	433 (20.3)	NA	0 (0.0)	433 (20.3)	NA
Very low	26 (3.2)	608 (28.4)	NA	0 (0.0)	608 (28.4)	NA	0 (0.0)	608 (28.4)	NA

*Values are no. (%) students unless otherwise noted. Continuous variables were compared by using a Kruskal-Wallis test; categorical variables were compared by using χ^2 test; Fisher exact test was applied when cell counts were <5 cells/μL. PCR-negative students were confirmed to have no malaria infection from any *Plasmodium* spp. IQR, interquartile range; NA, not applicable.
†Fever was defined as temperature ≥38°C at the time of survey. Temperature was missing for n = 618 (17.9%) students; percentages were calculated from nonmissing data.
‡Epidemiologic risk strata were defined according to *P. falciparum* prevalences in children from the 2014–15 School Malaria Parasitological Survey, Tanzania: very low if prevalence <5%, low if 5% to <10%, moderate if 10% to <50%, and high if ≥50%.
.

Appendix Table 8. Percentage of students with *P. ovale* spp. infections detected at PCR cycle thresholds between 40 and 45*

Cycle threshold	*P. ovale* spp., no. (%)
<40.0	75 (9.2)
40.0–40.9	93 (11.4)
41. 0–41. 9	203 (24.9)
42. 0–42. 9	228 (28.0)
43. 0–43. 9	158 (19.4)
44. 0–44. 9	57 (7.0)

*Total number of *P. ovale* spp. detected was 814.
